# Role of different non-coding RNAs as ovarian cancer biomarkers

**DOI:** 10.1186/s13048-022-01002-3

**Published:** 2022-06-17

**Authors:** Anam Beg, Rafat Parveen, Hassan Fouad, M. E. Yahia, Azza S. Hassanein

**Affiliations:** 1 Department of Computer Science, Jamia Millia Islamia, New Delhi, 110025 India; 2 Applied Medical Science Department, CC, King Saud University, P.O Box 10219, Riyadh, 11433 Saudi Arabia; 3 Faculty of Engineering and Natural Sciences, International University of Sarajevo, Sarajevo, Bosnia and Herzegovina; 4 Biomedical Engineering Department, Faculty of Engineering, Helwan University, Cairo, Egypt

**Keywords:** Long coding RNA, Ovarian cancer, Small non-coding-RNA, Gynecological malignancies, microRNAs (miRNAs), tRNA-derived small RNAs, Ovarian cancer

## Abstract

**Background:**

Among many gynecological malignancies ovarian cancer is the most prominent and leading cause of female mortality worldwide. Despite extensive research, the underlying cause of disease progression and pathology is still unknown. In the progression of ovarian cancer different non-coding RNAs have been recognized as important regulators. The biology of ovarian cancer which includes cancer initiation, progression, and dissemination is found to be regulated by different ncRNA. Clinically ncRNA shows high prognostic and diagnostic importance.

**Results:**

In this review, we prioritize the role of different non-coding RNA and their perspective in diagnosis as potential biomarkers in the case of ovarian cancer. Summary of some of the few miRNAs involved in epithelial ovarian cancer their expression and clinical features are being provided in the table. Also, in cancer cell proliferation, apoptosis, invasion, and migration abnormal expression of piRNAs are emerging as a crucial regulator hence the role of few piRNAs is being given. Both tRFs and tiRNAs play important roles in tumorigenesis and are promising diagnostic biomarkers and therapeutic targets for cancer. lncRNA has shown a leading role in malignant transformation and potential therapeutic value in ovarian cancer therapy.

**Conclusions:**

Hence in this review we demonstrated the role of different ncRNA that play an important role in serving strong potential as a therapeutic approach for the treatment of ovarian cancer.

## Introduction

Gynecological cancers, which include malignancies of the cervix, ovary, uterus, vulva, vaginal, and fallopian tubes, are among the top causes of female mortality around the world, with endometrial, ovarian, and cervical cancers being the most common. Ovarian malignancies, in particular, continue to be a major source of morbidity and mortality. Over the past four decades, only a few improvements have been made in the survival rates. The early stage of ovarian cancer is still curable in 90% of women, but a large number of women get symptoms when the disease progresses to the last stage. Based on histopathological features ovarian tumors are categorized into three subtypes i.e., epithelial, sex cords, and germline tumors. Among all OCs epithelial ovarian cancer accounts for 80-85% of ovarian cancer [1]. EOC is further classified into five major classes based on morphological and histological differences: High-grade serous carcinoma, Mucinous carcinoma, Low-grade serous carcinoma, Endometrial carcinoma, and Clear cell carcinoma. HGSOC was formerly believed to originate from the ovarian surface epithelium, however, new research suggests that the majority of advanced HGSOC comes from the fallopian tube fimbriae [2]. The most common and deadliest histotype of ovarian cancer is high-grade serous ovarian cancer (HGSOC), which accounts for about 75% of all EOC-related deaths [1]. TP53 is mutated in about 90% of individuals with the most frequent subtype, HGSOC. The development of a breakthrough therapy targeting poly ADP ribose polymerase (PARP) via inhibitors was prompted by advances in next-generation sequencing, which revealed that mutations in DNA repair pathways, including BRCA1 and BRCA2, are common in roughly 50% of HGSOC patients [3].

Pelvic examination, Transvaginal ultrasound, and measurement levels of CA125, HE4, p53, and Wnt/ β catenin are the few methods available for monitoring and detection of ovarian cancer. All these methods with few improved outcomes still lack adequate specificity and sensitivity. Hence for better management (prediction, progression, and response to treatment) of ovarian cancer, there is a need for new biomarkers. Bioinformatics paved the way for genome sequencing and gene expression profiling that are being used in clinical trials in the screening of potent biomarkers [4–6]. These methods are helpful to predict tumor molecular information, and chemotherapy prediction, and influence the survival rate of patients.

Today ongoing researchers have provided tools and techniques for improved diagnosis and management for advanced detection and effective targeted therapy with reduced toxicity. Hence for the regulation of tumor suppressors and oncogenes, research is being made to find suitable targets. Initially, 98% of genome sequences were allocated as junk DNA, which is further transcribed as non-coding RNA (ncRNA). These non-coding RNAs are important therapeutic targets because they modulate gene expression and disease progression [7]. Clinically ncRNA shows high prognostic and diagnostic importance. In this mini-review, we prioritize the role of different non-coding RNA and their perspective in diagnosis as potential biomarkers.

## Non-coding RNA as cancer Biomarkers

The well-known fact from the central dogma of molecular biology states that DNA is transcribed into RNA and RNA is translated into protein to mediate biological function. But to our surprise, the human genome project reveals that only 1.5% of the human genome encodes for protein-coding genes [7–9]. And the majority of RNAs in the human genome do not code for any protein and are therefore called non-coding RNA (ncRNA). Being heterogeneous in size it plays a significant role in the development of human disease. Small non-coding RNAs (ncRNAs) have been identified as essential regulators in ovarian cancer metastasis and progression. Their dysregulation affects gene expression and cellular signaling networks, and it’s seen in liquid biopsies.

**Fig. 1:**
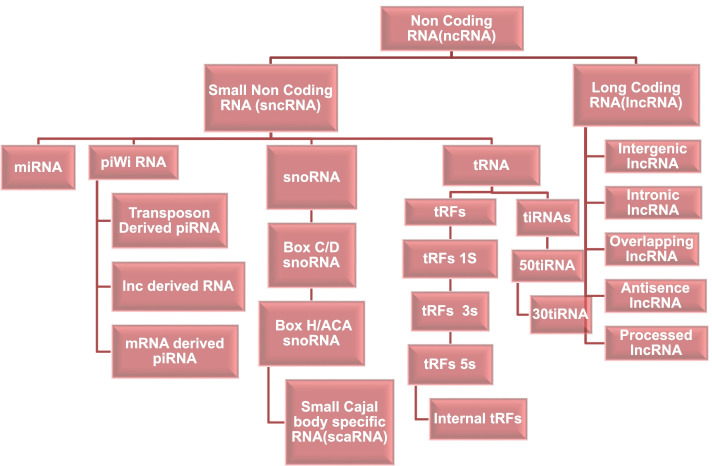
Different types of ncRNAs

A biomarker is a marker that can be used to predict clinical prognosis, risk of recurrence, or the diagnostic potential of a specific target. The development of new sequencing strategies; characterization of tumor pathways; high throughput screening of druggable targets and their predictive outcome leads to the success of biomarker-based therapy outcome [5, 6, 10]. One of the first biomarkers to reach clinical practice was the development and detection of mutations in the KRAS gene in cases of metastatic colorectal cancer (CRC) [11]. Hence a biomarker is a collection of genetic and proteomic signatures used to differentiate between diseased and healthy individuals. These signatures can be in the form of RNA (mRNA, miRNA, circRNA, and lncRNA), DNA (ssDNA, dsDNA, and retrotransposons), or protein (antibodies and peptides) depending upon the site of secretion and isolation. Based on their sizes ncRNA can be divided into two types, sncRNA (small non-coding RNAs) and lncRNA (long non-coding RNAs). Small non-coding RNAs are further divided into microRNAs (miRNAs), transfer RNA (tRNA), small nucleolar RNAs (snoRNAs), and piwiRNAs (piRNAs). Different types of ncRNAs are shown in Fig. 1.

The abnormal sncRNA expression has been linked to a variety of cellular dysfunctions and disease states. Multiple sncRNA groups appear to play essential roles in cancer start, development, and pathophysiology, according to growing evidence. sncRNA mutations have a strong diagnostic and prognostic value clinically. It is hoped that with a better understanding of the nature and functions of non-coding RNAs, we may be able to develop cancer therapies that act by modulating these RNA molecules. The development of medicines that may disrupt the oncogenic effects of non-coding RNAs has been inspired by advances in in-vivo nucleic acid delivery technologies and in-silico methodologies.

These ncRNAs are often deregulated in cancer and play a crucial role in different biological processes. Gene expression is regulated by miRNAs at the posttranscriptional level, whereas transcriptional and posttranscriptional events are regulated by lncRNA, and some play significant roles in the pathogenesis of human diseases. Many ncRNAs (lncRNA, siRNA, miRNA, piRNA, and circRNA) play significant roles in various stages of cancer; their roles are well demonstrated in ovarian cancer and its treatment [12, 13]. Necrotic and apoptotic cells help [14] them to get released into blood circulation where they are associated with proteins called argonaute [12, 13, 15, 16]. Argonautes are helpful to ncRNAs, particularly these proteins bind to short non-coding RNAs and play a significant role in RNA-based silencing by regulating protein synthesis and RNA stability. It also helps to maintain chromosome integrity, further, it is involved in siRNA and miRNA maturation, and can even contribute to the generation of Piwi-interacting (pi)RNAs, a new class of short non-coding RNAs [17].

ncRNAs are considered suitable biomarkers for detecting many human diseases including cancer. They can easily differentiate between normal healthy individuals from patients, they are stable in circulation, they can be easily collected through non-invasive methods, and reflect disease progression. All these properties fit very well in ncRNA, especially circulating miRNAs. Quantitative RT-PCR or high-throughput techniques like nanostring or miRNA microarrays can readily determine the level of circulating miRNAs. Chao et al. investigated potential miRNAs in the serum of patients with clear cell OC employing quantitative PCR (qPCR) for 270 miRNAs. Before the serum marker CA125 was revealed to be high, they discovered elevated serum levels of miR-130a in early illness recurrence [18]. More interestingly, the accuracy of profilinnong specific miRNAs rather than traditional platelet counts, prothrombin international normalized ratios (INRs), or albumin in predicting illness appears to be quite significant [19]. As a result, specific miRNA expression profiles in sera, when combined with biochemical data, may be more effective non-invasive predictive and therapeutic markers for ovarian cancer. Hence to assess the miRNA measurement for biomarkers serum, plasma and ascites are used. Also, lncRNAs are a diverse group of ncRNAs that can be used as diagnostic and prognostic biomarkers in cancer. Circulating lncRNAs have proven to be incredibly useful in the identification of several cancer types in recent years. They are useful as biomarkers not only because circulating lncRNAs may be acquired readily and non-invasively from cancer patients, but also because these lncRNAs are relatively persistent in body fluids. Besides these, a list of Circulating ncRNAs involved in advanced ovarian cancer had already been described by Schwarzenbach and Gahan. In this paper,r we will mention the role of only those ncRNAs that are involved in ovarian cancer.

### Different types of sncRNA involved in ovarian cancer

#### miRNA

miRNAs is a small (18-25 nucleotides in length) single-stranded non-coding RNA that helps in gene expression (RNA silencing and post-transcriptional regulation). The majority of genes coded by proteins are controlled by miRNA. With help of northern blotting and q-PCR researchers can identify miRNA in body fluids and differentiate between miRNA isomers respectively. But since these methods lack sensitivity, it is, therefore, sequencing of small RNAs to identify novel RNA. Fifteen years ago, since the first evidence of miRNA came it was established that miRNA plays an important role in the evolution and progression of cancer. Hence dysregulation in signatures of miRNAs is involved in every stage of ovarian cancer. So now we affirm that signatures of miRNA can differentiate between tissues of normal and cancer subtypes, in various cancers because miRNAs are tissue-specific, they are disrupted in a variety of disorders. However, because they are secreted in body fluids, some miRNAs are promising candidates for biomarker research. In comparison to mRNA expression signatures, miRNA signatures are shown to have more predictive power [20, 21]. In ovarian physiology Deregulation of miRNA in OC is an important feature for its regulation. In epithelial ovarian cancers (EOC) miR-29b, miR-125b, miR-29a, and let-7 are downregulated and are exceptionally expressed in normal ovarian tissues. In normal ovarian functioning, this deregulation of miRNA is now linked to EOC pathogenesis by the researchers. For instance, 39 miRNAs in tumor tissues are remarkably deregulated in contrast to the normal ovary, out of which the overexpressed miRNAs are miR-141, miR-200a, miR200c, and miR200b on the other hand down-regulated miRNAs were miR-199a, miR-140, miR-145 and miR-125b1 [22]. The emphasis on miRNA networks in EOC survival is shown in various studies which conclude that eight miRNAs (miR-101, miR-141, miR-506, miR-25, miR-29c, miR-182, miR-128, and miR-200a) that were downregulated were predicted to regulate the majority of miRNA-associated genes [20, 21, 23]. Table 1 describes the Summary of some of the few miRNAs involved in epithelial ovarian cancer their expression and clinical features.

**Table 1:** Features different types of miRNA, their expression pattern, signaling pathways, functions, and target regulators

miRNA	Expression Pattern and No of Clinical Samples	Signaling pathways	Target Regulators	Function	References
miRNA-1	Downregulated 5 non-neoplastic ovarian tissues and 13 ovarian cancer specimens	Akt, ERK1/2	c-met	miRNA-1 takes part in tumor suppression by inhibiting the expression of c-met	[24]
miRNA-124	Downregulated 13 pairings of adjacent non-tumor tissues and ovarian cancer tissues	–	SphK1	Downregulation of SphK1 is supported by miR-124 which suppresses migration and invasion of Ovarian Cancer	[25]
miRNA-335	Downregulated ovarian cancer tissues and non-tumor tissues in ten pairs	–	Bcl-W	In epithelial ovarian cancer, miR-335 is responsible for poor survival	[26]
miRNA-215	Downregulate A total of 48 pairs of ovarian cancer tissues and non-tumor tissues were studied.	MAPK	NOB1	NOB1is helpful in inhibiting the growth of tumor cells with the help of miR-215	[27]
miRNA-429	Downregulated A total of 72 pairs of ovarian cancer tissues and non-tumor tissue were used in this study.	–	ZEB1	Cisplatin sensitivity increases due to Overexpression of miR-429 which help in reducing autophagy-related protein anti-LC3A/Band Anti-ATG7	[28]
miRNA-200c	Upregulated	–	ZEB1	Inhibition of EMT in CD44 + CD117 + CSCs is supported by miR-200c overexpression which decreases ZEB1 expression.	[29]
miRNA-7	Upregulated 180 samples of ovarian cancer, 66 samples of normal ovarian tissue	AKT, ERK1	–	The overexpression of miR-7 in SKOV3 cells increases cell invasion and migration.	[22]
miRNA-25 miRNA-93	Downregulated 180 samples of ovarian cancer, 66 samples of normal ovarian tissue	AKT/ERK1	–	In SKOV3 cells, overexpression of miR-7 increases cell migration and invasion.	[22]
miRNA-137	Downregulated There were 21 cases of serous adenocystic carcinoma, 14 cases of mucinous adenocystic carcinoma, and 29 cases of normal ovarian tissues.	–	XIAP	miR-137 promotes Epithelial Ovarian cells to undergo cisplatin-induced apoptosis.	[30]
miRNA-490-3p	Downregulated 139 samples of ovarian cancer, 17 samples of normal ovarian tissue		CDK1	Induced expression of p53 Cell cycle phases G1/SorG2/arrest and apoptosis is promoted by overexpression of MiR-490-3P and reduces cell invasion, migration, proliferation, and invasion.	[31]
miRNA-145	Downregulated 38 samples of ovarian cancer, 9 samples of normal ovarian tissue	–	TRIM2	miR-145 mediates inhibition of TRIM2 which leads to the up-regulation.	[32]
miRNA-92	- 3 pairs of ovarian cancer tissues and non-cancer tissues adjacent	Wnt/β-catenin, STAT3	DKK1	In epithelial ovarian cancer, the STAT3-miR-92a-DKK1 pathway has potential therapeutic applications for targeted therapy	[33]
let-7d-5p	Downregulated 76 pairings of non-tumor tissues and ovarian cancer tissues	p53	HMGA1	The proliferation in ovarian cancer cells is suppressed and chemosensitivity is restored through the let-7d-5p by activating the p53 signaling pathway and silencing HMGA1.	[34]
miRNA-206	Downregulated 50 samples of ovarian cancer tissues	AKT/mTOR	c-Met	mir-206 could suppressor through c-Met/AKT/ mTOR signaling pathway	[35]
miRNA-630	Upregulated	–	APAF-1	miR-630 targets APAF-1 in re-sensitizing the cells to chemotherapy.	[36]
miRNA-152	Downregulated 8 pairs of cancer tissues and adjacent non-cancerous tissues	–	FOXP1	The proliferation and migration in epithelial ovarian cancer cells are inhibited by miR-152 by targeting FOXP1.	[37]
miRNA-142-3p	Downregulated There are 58 pairs of malignant and non-cancerous tissues in this study.	–	Sirtuin 1	The proliferation and chemoresistance in ovarian cancer could be inhibited by miR-142-3p by targeting SIRT1.	[38]
miRNA-221	Upregulated There are 63 pairs of cancer tissues and surrounding non-tumor tissues in this study.	–	APAF-1	The proliferation of cells in OC is promoted by overexpression of miRNA-221 by targeting APAF 1.	[39]
miRNA-145-5p	Downregulated -	Hippo	CTGF	Lower expression of miR-145-5p in Ovarian cancer is reviewed as a biomarker for diagnosis.	[40]
miRNA—199b-5p	Downregulated 79 EOC patient	Notch1	JAG1	Acquired chemoresistance in ovarian cancer is associated with miR-199b-5p via activating JAG1/ Notch1 signaling.	[41]
miRNA-141	Upregulated 132 fresh-frozen ovarian cancer samples	NF-kB	p38a MAPK, YAP1, KEAP1	In ovarian cancer cell lines, miR-141 overexpression can increase resistance to cisplatin. It also modulates cisplatin sensitivity by targeting KEAP1.	[42]
miRNA-424-5p	Downregulated surgical resection of 83 primary and 19 matched normal tissues	E2F1-pRB	CCNE1	G0/G1 cell cycle arrest and proliferation of ovarian cancer cells are being done miR-424-5p by targeting CCNE1 mediated E2F1- pRb signaling pathway.	[43]

#### piRNA

These are single-stranded non-coding RNAs of 26-31 nucleotides in length. In the silencing of retrotransposons, piRNAs play an important role in epigenetic and post-transcriptional levels. Based on origin piRNAs can be divided into (a) transposon-derived piRNA, (b) lncRNA-derived piRNAs, and (c) mRNA-derived piRNAs. All these piRNAs are gathered on chromosomes two, four, five, and seven on short genomic loci. RNA polymerase II transcribed piRNAs which are then transported to the cytoplasm and prepared into smaller sequences (mature piRNAs). Although the function and biogenesis of piRNAs are not clear, studies with deep sequencing technology help to compare different expression profiles of these sncRNAs in different tissues. Particularly, researchers now search for differential expression of different sncRNAs in tumor tissues to compare with normal tissues in different metastatic cancers [3, 12, 13, 44].

Researchers showed that PIWI proteins are also associated with cancer hallmarks. piRNAs also show a distinguished role in OC. Four PIWI proteins i.e., PIWIL1/HIWI, PIWIL2/HILI PIWIL3/HIWI3 and PIWIL4/HIWI2 that have been identified in the prognosis and diagnosis of Ovarian Cancer [12, 13, 45]. Weng, Li et al. reviewed piRNAs’ involvement in prognosis, diagnosis, migration, and invasion in different cancers [46].

piRNAs are the advancing important regulator in the proliferation, apoptosis, invasion, and migration of cancer cells [3]. The study and expression of piRNA in various gynecological malignancies remains exploratory. To detect piRNAs in various samples including normal ovary (pi RNA #219), serous ovarian cancer (pi RNA #234), and endometrioid (pi RNA #256), Singh et al. used the method of RNA sequencing [47]. The study involved small sample numbers but still, authors reported around 143(56 upregulated and 81 downregulated differentially expressed piRNAs) and 159 (74 upregulated and 77 down-regulated, differentially expressed piRNAs) differentially expressed piRNAs in serous ovarian cancer and endometrioid respectively. Extensive research concludes piR-52,207 was found to be upregulated in endometrioid ovarian cancer, and piR-52,207 and piR-33,733 were increased in serous ovarian cancer [47]. piR-52,207 and piR-33,733 promote endometrioid ovarian cancer and serous ovarian cancer cell proliferation, migration, and tumorigenesis respectively. This is being done by targeting MTR, NUDT4, EIF2S3, and MPHOSPH8 by piR-52,207 and LIAS30-UTRs by piR-33,733 respectively. At the post-transcriptional level, piR-52,207 and piR-33,733 enhance ovarian cancer oncogenes through their involvement in several cell-signaling pathways, suggesting that they could be used as drug targets in ovarian cancer [3, 47].

#### tRNA (tRNA-derived small RNAs)

Transfer RNA is the most prevalent cellular ncRNA that is crucial for protein translation (tRNA). Depending on the cleavage site, tsRNAs can be divided into two groups the first one is tRFs (transfer RNA-derived RNA fragments) which are derived from mature tRNAs and is 14 to 30 nucleotides in length. The second type is tRNA halves, or tiRNAs, which have a length of 29 to 50 nucleotides and are generated by stress. It is produced by specific cleavage at the anticodon loop of mature tRNA. tRFs are further categorised into tRF-1 s, tRF-3 s, tRF-5 s, and internal tRFs (i-tRFs or tRF-2 s), while tiRNAs are subdivided into 50tiRNA and 30tiRNA. Both tRFs and tiRNAs play essential roles in carcinogenesis and have the potential to be used as predictive novel therapies targets in cancer [3]. tRFs interact with aminoacyl tRNA synthetases and ribosomes together with this they can modulate protein translation. Additionally, tRFs may influence gene expression by forming cell-type-specific associations with Ago and PIWI proteins. Furthermore, the association between RNA-binding proteins and tRFs is important in cancer development and metastasis. tsRNAs drive cell cycle progression and proliferation in many malignancies by influencing proto-oncogene and oncogene expression levels [48].

One of the tRFs has been found to play a significant role in ovarian cancer i.e., tRF5Glu [3, 49]. The expression levels of two different tRFs i.e. ts-101 and ts-46 (tRF-1 s) correlate with cell survival, apoptosis, cell proliferation, chromatin structure, and clonal growth, in ovarian cancer, colon cancer, and breast, cancer patients, as well as corresponding cell lines. In oncogene activation and progression of ovarian cancer the expression of tRFs plays a significant role [50]. In around 180 serum samples which includes 97 patients with ovarian cancer, 22 borderline tumors, 15 healthy controls, and 46 benign tumors; it was revealed after reanalysis of RNA-sequencing data, that out of total small RNA, tsRNAs make up a large part of serum samples, ranging from 2.5–29.4%, and they are enriched in a plethora of different tRNA (e.g., Gly-tRNA), as well as being able to accurately detect aberrant cell proliferation [51]. In a study using blood samples from ovarian cancer patients and healthy donors, as well as ovarian cancer cell lines, it was discovered that tRF-03357 inhibits HMBOX1 and promotes SK-OV-3 cell migration, proliferation, and invasion [48].

### Long non-coding RNA (lncRNA) involved in ovarian cancer

lncRNA is the RNA transcript with a length exceeding 200 nucleotides which are not translated into proteins. Neither do they have any open reading frames nor do they code for any proteins (But still there is a debate between some authors who showed they do encode for protein). In terms of protein-coding genes, and based on location lncRNAs are classified as (1) intergenic lncRNAs that are present between two protein-coding genes (2) intronic lncRNAs introns of protein-coding genes transcribe them), (3) overlapping lncRNAs a coding gene is located on the intron (4) antisense lncRNAs-the opposite strand of protein-coding gene transcribes them (5) processed lncRNAs - lacks an open reading frame ORF. Ren et al. found about 210,000 lncRNAs, of which 106,063 are humanly related (lncRNAWiki, 2015), while only 16,840 were found in Gencode (https://www.gencodegenes.org) [52]. RNA polymerase II transcribes lncRNAs, which are regulated by the SWI/SNF complex’s transcriptional activators. Gene transcription, imprinting, X-chromosome inactivation, nuclear architectural organization, and epigenetic chromatin modification are a few of the complex functions that lncRNAs perform [20, 21, 53]. Multiple processes involving lncRNAs can play a role in cancer development and progression. The role of lncRNAs in cancer progression has been intensively researched, primarily via epigenetic regulation, triggering of carcinogenic pathways, and interaction with other RNA subtypes. Some lncRNAs, may be isolated non-invasively from the blood and are highly tissue-specific and abnormally expressed in cancer. Using lncRNA as markers has an advantage over protein-coding RNAs because their expression is a better indication of the tumor state. Because of new technology, several lncRNAs are now linked to cancer and are emerging as novel regulatory players in molecular biology. lncRNA often has tissue-specific patterns that differentiate them from miRNAs and protein-coding mRNAs, which are expressed in a variety of tissues [7]. They are precise biomarkers for cancer diagnoses because of their specificity [7, 54]. These characteristics make them prospective candidates for cancer detection. PCA3, a biomarker for early prostate cancer diagnosis, is the most well-known (PCa).

Certain forms of highly expressed cancer-associated lncRNAs can be used to find new potential biomarkers [55]. Pathways mediating transcriptional gene silencing, particularly those of tumor suppressors and oncogenes, can provide therapeutic benefits [56]. Biomarkers should be detected in non-invasive samples for the comfort of patients. Body fluids, such as serum or urine, are desirable samples because they contain circulating nucleic acids (CNAs), both DNA and RNA species. Plasma, cell-free serum, sputum, and urine all contain CNAs [57, 58]. Due to high stability while circulating in body fluids that makes lncRNAs are suitable for cancer diagnostic and prognostic biomarkers especially when included in exosomes or apoptotic bodies [59, 60]. Table 2 emphasized those lncRNAs that have shown a leading role in malignant transformation and potential therapeutic value in ovarian cancer therapy.

**Table 2:** Depicts the clinical significance of different lncRNAs in ovarian cancer

lncRNAs in ovarian cancer	Location	Length	Clinical Significance	Reference
**H19**	Chr11p15.5	2.3	In ovarian cancer, when H19 is highly expressed it triggers migration and invasion of tumor cells	[61]
**HOTAIR**	Chr12.q13.13	2.2	HOTAIR plays crucial role in chemoresistance and its increased sensitivity towards cisplatin causes autophagy in OC	[62]
**X inactivate-specific transcript (XIST)**	ChrX	17	XIST is found to be a potent biomarker for patients who respond to first-line chemotherapy because a high affinity is found between regulation of XIST and patient response to chemotherapy using paclitaxel.	[63]
**UCA1**	Chr19p13.12	1439	UCA1 is a dominant biomarker for several cancer types. Up-regulation of UCA1 is linked with progression-free survival (PFS) in ovarian cancer patients.	(Hong, Hou [64])
**MALAT-1**	Chr11q13	> 8000 nucleotides	Wnt/β-catenin signaling pathway is affected by; HOTAIR, MALAT-1 is, therefore, downregulation of MALAT-1 is used to inhibit OC cell viability, migration, and invasion,	[65]
**Plasmacytoma variant translocation 1 (PVT1) PVT1**	Chr8q24.21	1716 nucleotides	Progression of ovarian cancer is supported by PVT1 by silencing miR-214.	[66]
**GASS**	1q25.1.10	0.7	The expression of GAS5 is more prevalent in EOC than in normal ovarian epithelium tissue.	[67]
**Homeobox protein D cluster antisense RNA 1 (HOXD-AS1**	Chr2q31.2		Raised levels of HOXD-AS1 were adversely correlated with PFS and OS of EOC patients.	[68]
**Sprouty RTK signaling antagonist (SPRY4-IT1)**	Chr5q31.3	708	Progression of ovarian cancer might occur due to SPRY4-IT1 downregulation [119]. But still, the mechanism of this long codinglncRNAA is complicated, whether it acts as a tumor suppressor or an oncogene in ovarian tissue	[69]

## Future perspective

PIWI-like (PIWIL) genes, which belong to the Ago family, are frequently altered in ovarian cancer, and these proteins, along with piRNA, are implicated in several stages of malignancy and are linked to advanced tumor stage and poor prognosis. The PIWI proteins, together with piRNA, are primarily expressed in cancer cells, making them excellent indicators for cancer diagnosis as well as potential therapeutic targets.

tsRNAs are reported to be dysregulated in a variety of cancers, including gynecologic malignancies, and because Ago proteins (like miR) and PIWI proteins (like piRNAs) are accompanied by these tsRNAs, they can influence gene expression both pre-and post-transcriptionally (like piRNA) (like miR). Since tsRNAs may be easily found in cancer patients’ urine and serum, they are considered powerful diagnostic indicators. As a result, because tsRNAs are involved in tumor initiation, progression, and medication response, they could be used as therapeutic targets or diagnostic markers. Our comprehension of the roles of piRNA- and tRNA-derived small RNAs is still limited; however, much valuable information is available for miR, and a few studies are in the early stages of development for cancer therapy, with some clinical studies in headway for ovarian cancer e.g. (ClinicalTrials.gov Identifier: NCT03738319, NCT03776630, NCT01970696, NCT02253251, NCT03877796). Since each trial has been assigned with a number hence the details of each trial (e.g., Study type, Number of participants involved in the study, the start date of the study, primary completion date, study completion date, etc) can be found at ClinicalTrials.gov Identifier. Based on this growing body of research, it’s feasible that miRs in plasma will be utilized to diagnose the prognostic malignancies shortly. On the other hand, the abnormal expression and involvement in a variety of biological functions, lncRNAs could be used to treat cancer. In an assorted lineage of cancer, lncRNAs are either aberrantly expressed or mutated [70]. Several lncRNAs have been demonstrated to have an inhibitory or stimulating effect on tumor formation, metastasis, cell survival, immunological response, and chemoresistance in ovarian cancer [71–73]. Since lncRNAs play a pivotal role in ovarian cancer, they can be used as prognostic and diagnostic markers as well as therapeutic targets. For instance; 53 lncRNAs associated with ovarian cancer have been revealed by extensive RNA sequencing. Only 25 are directly related to a clinical purpose, whereas 27 were upregulated and 26 were downregulated [74]. Even from these few lncRNAs, it is evident that additional research is needed to link them to a specific stage in the development and management of OC to determine their specific roles in tumor formation, cell migration, and metastasis. The importance of deciphering the processes of lncRNA has already been established in clinical investigations. Even though certain lncRNAs have been thoroughly studied in clinical trials or patented, their applications still have a long way to go.

## Conclusion

Due to the heterogeneity of the illness, a patient with ovarian cancer has several significant problems, including late detection, poor prognosis, a lack of accurate indicators, and resistance to available treatments. Considering a substantial contribution to the study of sncRNA, it is now well accepted that these small non-coding RNAs play a pivotal role in gene regulation and play a crucial part in the development of Ovarian cancer. A single small non-coding RNA can distinguish between diseases; however, a combination of sncRNA indicators could be illustrative of the molecular progression of ovarian cancer for early diagnosis and increased disease monitoring. Novel and well-studied sncRNAs are required for a better understanding of disease progression. As a result, new approaches are required to measure these alterations and enable the development of future biomarkers.

Over the last 20 years, it has been made clear how miRNA regulates different hallmarks of cancer and now it’s our priority to take advantage of this knowledge to improve cancer patient diagnosis and prognosis. However, there are still several obstacles that stand in the way of using miRNA as a diagnostic and prognostic biomarker, including genetic background, sample procedures, detecting techniques, ethnicity, and genetic background. To overcome the foregoing factors on the observed miRNA signatures, more investigations in larger patients are needed. The study of miRNA regulation in ovarian cancer is still in its early stages, accounting for only 4% of published studies. The majority of that 4% of published publications deal with target gene discovery, expression profiling, determining affected cellular pathways, resistance to traditional chemotherapeutics, and their use as therapeutic tools. As a result, we demonstrated the function of distinct sncRNAs in this review, which have a lot of potential as a therapeutic method for ovarian cancer treatment.

## Data Availability

In the current study as such there is no datasets used.

## Abbreviations

OC: Ovarian Cancer
sncRNA: Small Non-Coding RNA
lncRNA: Long Non-Coding RNA
tRNA: Transfer RNA
miRNAs: Micro RNA
piRNA: Piwi-interacting RNA
tiRNA: tRNA-Derived Stress-Induced RNAs
miR: MicroRNAs
tRFs: Transfer RNA-derived RNA fragments
CA125: Carbohydrate antigen125
HE4: Human epididymis protein 4
